# Gender Differences in Mother-Child Conversations About Shame and Pride in a Hungarian Sample

**DOI:** 10.5964/ejop.2859

**Published:** 2021-05-31

**Authors:** Melinda Pohárnok, András Láng

**Affiliations:** a Department of Developmental and Clinical Psychology, Institute of Psychology, University of Pécs, Pécs, Hungary; University of Neuchâtel, Neuchâtel, Switzerland

**Keywords:** parent-child conversations, pride, shame, gendered socialization of emotions, conversational style, emotion words

## Abstract

Although meta-analytic reviews repeatedly found significant gender differences in the experiences of shame and pride throughout the life span, to date, gender differences in conversations about these emotions have not been studied. Our research was aimed at investigating the effect of child gender on maternal conversational style in and emotional content of mother-child conversations about shame- and pride-related past events in preschool years. Fifty four mother—preschool child dyads (52% girls, children’s age M = 70.36 months [SD = 8.13], mothers’ age M = 37.51 years [SD = 3.70]) from middle class Hungarian families were asked to talk about two past events, one in which children felt ashamed, and one in which they felt proud. The conversations were transcribed and coded for maternal conversational style and for emotional content. Maternal conversational style was indicated by maternal elaboration and evaluation of the child’s contributions. Emotional content was indicated by specific emotion terms, emotional behavior and emotional evaluations. In mother-son shame conversations, we found higher amount of negative emotional behavior. Boys also had longer conversations with their mothers, and mothers used more open-ended memory questions and more repetitions with boys in both shame and pride conversations. Girls had shorter contributions to pride stories than to shame stories, which was not the case for boys. Exploration of verbal socialization of shame and pride helps us to understand the development of individual differences in proneness to self-conscious emotions, and their implications for mental health.

One arena of emotion socialization in the preschool period is the parent-child discussion of emotion-related events (Brody, 1999). Several studies found gender related differences in talking about basic negative and positive emotions, which influence gendered expression and experience of emotions later on (Fivush & Grysman, 2019 for a review). However, we are ignorant of any studies investigating gender differences in conversations about shame and pride in this salient age range for gender and emotion socialization. Our purpose was to investigate gender-related differences in mother-child conversations about shame and pride, which might have implications for gender-differences in the experiences and in the expression of these emotions in later childhood and adulthood (Chaplin & Aldao, 2013; Else-Quest, Higgins, Allison, & Morton, 2012).

## Shame and Pride

Shame and pride—similarly to other self-conscious emotions, such as guilt and embarrassment—are human-specific emotional experiences that are evoked by self-reflection and self-appraisal (Tangney & Tracy, 2012). Guilt and shame are both experienced in situations in which behavioral and/or moral standards are violated, and the individual expects that others will disapprove their action resulting in rejection or losing face (Tangney & Tracy, 2012). Although these emotions are often referred to (and experienced) as interchangeable, theorists suggested several aspects by which they could be differentiated. Lewis (1971) indicated that guilt is concerned with the negative evaluation of a specific behavior, and shame derives from the negative evaluation of the global self. Olthof, Ferguson, Bloemers, and Deij (2004) assumed that shame is linked to the threat of unwanted identity, and guilt arises from the individual’s perception of being involved in the causation of moral wrong.

Pride is evoked if we perceive that we achieved a socially valued outcome and we are valued as a person (Mascolo & Fischer, 1995). Thereby pride not only enhances one’s self-worth, but maintains or even improves the person’s position in the social hierarchy (see Tangney & Tracey, 2012).

With the advance of theory of mind children become increasingly aware of the fact that parents and significant others have expectations regarding their behavior, others evaluate them from an external perspective and they can generate emotional states in these others. This makes children more prone to experience guilt, shame and pride (Lagattuta & Thompson, 2007). Lewis (2000) argued that beside conceiving of social rejection and acceptance, children also require the capacity of self-awareness—the ability to form a stable self-representation—and an increasing awareness of rules and norms to be able to experience self-conscious emotions.

Since complex cognitive attributional process and cognitive self-awareness are essential to experience these emotions, the precursors and early manifestations of guilt, shame and pride cannot be seen earlier than 30–36 months (Kochanska, Gross, Lin, & Nichols, 2002; Tracy, Robins, & Lagattuta, 2005). It should also be noted that children become only progressively capable of differentiating negative self-conscious emotions, and they become able to differentiate the meaning of the terms “guilt” and “shame” only from the end of the preschool period (Olthof, Schouten, Kuiper, Stegge, & Jennekens-Schinkel, 2000).

## Gendered Socialization of Shame and Pride

Emotional experience and emotional response are organized and modulated by gender related social norms and culture-specific beliefs about the appropriate and valued responses associated with affections in a given culture (Ellemers, 2018). In Western culture, women are stereotyped as experiencing more guilt, shame, and embarrassment, but less pride than men (Plant, Hyde, Keltner, & Devine, 2000). Several research findings (e.g., Ferguson & Crowley, 1997; Tangney, 1994) support the presumption that women are encouraged and therefore more liable to experience guilt and shame. Ferguson and Eyre (2000) argued that the socialization process, situational pressures, and the roles of men and women in the social hierarchy could affect women’s higher report of shame and guilt. Gender roles and stereotypes suggest that women and girls are more interpersonally sensitive and therefore more threatened by the loss of love and more dependent on others' approvals (Lewis, 1978; cit. Ferguson & Eyre, 2000). Given this communal orientation of women and the fact that guilt arises from the individual’s belief that her behavior disadvantaged a loved one, it is not surprising that women are more susceptible to believe that they disadvantaged others without an acceptable reason. On the other hand, the traditional female roles are associated with lower social status and with interpersonal submission over dominance (Ellemers, 2018). This may lead women to report (and/or experience) more shame than men, because of their feelings of passivity and helplessness that are internalized through gender-specific socialization. In contrast, men are seen through the lens of gender stereotypes as more achievement-oriented, autonomous and agentive, and as generally having greater status and power in relationships than women (Haines, Deaux, & Lofaro, 2016). Consequently, it can be expected that men would report lower guilt and shame, because disadvantaging others is more accepted from men in their competitive relationships. Moreover, because of their higher status, men are less probable to encounter the threat of devaluation.

Ferguson and Eyre (2000) summarized some supportive evidence that these differences are deeply rooted in the gender related socialization of self-conscious emotions. During the childhood years parents and other socialization agents emphasize girls’ responsibility and blameworthiness for others' well-being, and girls receive more person-oriented and psychologically oriented inductions, that are aimed at facilitating empathy and responsibility for other’s feelings (Zahn-Waxler, 1993; Zahn-Waxler & Robinson, 1995). In a study examining parents’ evaluative behavior toward their 3 years old children in a problem-solving situation, Alessandri and Lewis (1993) found that parents gave more positive evaluations to boys and more negative evaluations to girls. Mills, Arbeau, Lall, and De Jaeger (2010) found that girls experienced more frequent shaming especially from mothers that increased with age between preschool and school age.

Current meta-analytic studies with different age groups point to the outcome of the previously mentioned socialization effects with finding that in both children and adult samples females experience more shame and guilt than men. Else-Quest et al. (2012) aimed to investigate the extent of gender differences in self-conscious experience and found that these gender differences were small but significant with regard to shame and guilt. Studies investigating younger age groups—preschoolers, school-aged children, and adolescents—produced similar results. Chaplin and Aldao (2013) in their comprehensive meta-analytic review of gender differences found that girls showed more shame expression than boys especially in novel situations with non-familiar adults. In another study (Mills et al., 2010) exploring shame responding in childhood and shame proneness in middle childhood, girls showed more shame than boys by school age. Finally, girls—compared with boys—displayed higher levels of negative self-conscious emotions in a scenario-based study of shame and guilt at 9–13 years and at 13–18 years as well (Muris et al., 2014).

Whereas there are small but consistent gender gaps in experiencing and expressing shame and guilt, the previously mentioned meta-analytic studies (Chaplin & Aldao, 2013; Else-Quest et al., 2012) did not find any significant differences between males and females in the experience of pride. In a qualitative review, Brody (1999) described boys to minimize emotional expressions with some important exceptions: notably anger, pride, and contempt. Men were also found to report more pride and feelings of success than women (Brody, 1999). In theorizing about the gender differences in positive emotions, Alexander and Wood (2000) presumed that men experienced more self-oriented positive emotions (e.g., pride for own success) and women were more probable to feel other oriented positive emotions (e.g., joy for others’ success).

## Parent-Child Conversations About Emotional Events

Parent-child conversations about emotions and past emotional events are arenas in which children acquire how to express and modulate their emotions, what kind of situational and behavioral antecedents and consequences typical emotions have (Denham, Bassett, & Wyatt, 2010). What and how children learn about emotion are influenced by gender-related cultural norms that determine the appropriateness of emotional experiences and displays for males and females (Root & Denham, 2010). Fivush (1989) proposed that in discussing past emotional events with children, mothers may encourage boys to engage in problem-solving and emotional control, and may encourage girls to focus on emotional states and sensitivity. Parents’ socialization efforts aimed at facilitating detailed verbal expression of emotions in girls may be associated with a general stereotype in Western culture, namely that reminiscing, especially emotional reminiscing is a stereotypically female activity (Fivush & Zaman, 2014).

Individual differences in parental conversational activity and facilitation can be conceptualized through elaboration. Highly elaborative parents use many questions and statements that add new information to the ongoing narrative, and provide a great deal of evaluative feedback to the child, namely confirm and repeat the child’s utterances (Reese & Fivush, 1993). By confirming and praising their children, highly elaborative parents both encourage their children's participation and convey that this involvement is valued (Fivush, Haden, & Reese, 2006). As a result, a more elaborative parental conversational style will lead to greater emotional understanding in children (Laible, 2004a). Studies found that mothers generally talk more and in a more elaborative conversational style about emotions with their daughters than with their sons (e.g., Fivush, Berlin, McDermott Sales, Mennuti-Washburn, & Cassidy, 2003). This is especially true for submissive negative emotions: mothers discuss the emotional aspects of sad events more frequently with their daughters than with their sons (Fivush, Brotman, Buckner, & Goodman, 2000). Moreover, mothers use a more elaborative conversational style when discussing sadness with their daughters—as compared to discussions of sadness with sons (Reese & Fivush, 1993; Sales, Fivush, & Peterson, 2003). By contrast, stories involving anger and moral transgressions are discussed in a more elaborative way with sons as compared to daughters (Bird & Reese, 2006; Fivush, 1989). Therefore, it might be concluded that mothers may encourage boys to have an elaborated understanding of anger and girls to have an elaborated understanding of sadness. This is in line with studies that suggest sadness to be a stereotypically female and anger to be a stereotypically male emotion (see Plant et al., 2000 for a review).

Some studies however (e.g., Laible, 2004b; Laible, Panfile Murphy, & Augustine, 2013) did not find significant associations between children’s gender and the quality of mother-child reminiscing, or found gender differences that were independent from the emotion discussed. In the studies of Sales et al. (2003) and Fivush et al. (2003), mothers were more elaborative with daughters, than with sons when discussing both positively and negatively valenced experience. This means that mothers more frequently told and asked for enriching new details in conversations with daughters and confirmed, negated or repeated girls’ utterances more frequently, but asked sons more close-ended questions—asked them to confirm or deny new information. Authors assumed that boys’ decreased willingness and/or capacity to participate in reminiscing made mothers provide more structured questions to keep boys engaged.

It could also be presumed that not just the conversational style of discussions about past events would be affected by the gender of the child, but the emotional content (i.e., the frequency of words or phrases referring to specific emotions or emotional behavior), and evaluative language would differ for boys and girls as well. However, research results are mixed. Although Fivush et al. (2000) found that parents discussed the causes of sadness in more length with daughters than with sons, they did not find gender differences in discussing the causes of happy, angry or scared feelings, nor in the explicit use of emotion words in emotion conversations. Bird and Reese (2006) found that in past event conversations girls referred more often to their negative emotions and used more external evaluation of themselves or other people. Similarly, the previously mentioned study of Fivush et al. (2000) showed that girls used more emotion words when talking about scary events. In contrast, Laible et al. (2013) found that gender was not significantly correlated with maternal or child discussion of emotions, causes of emotions and coping when mothers and their 42 to 48 months old children discussed an event in which the child experienced a negative emotion.

These contradictory results could be explained if we take into consideration the analyzed aspect of reminiscing, the age group under investigation, and the intensity of the emotion discussed. For example, in a study applying a detailed coding of conversational style, Reese and Fivush (1993) found that mothers are more elaborative with daughters than with sons between 40 and 70 months of age, but using a global coding of elaboration, Zaman and Fivush (2013) did not find differences in maternal elaborative style as a function of child’s gender. In the use of emotion words in the discussion of sad events, Adams, Kuebli, Boyle, and Fivush (1995) found that there were significant differences favoring daughters at 40 months, but there were no differences at 70 months of age. The intensity of distress might also influence the effect of gender. When discussing a highly emotional event—an emergency room visit (Peterson, Sales, Reese, & Fivush, 2006) or intensely positive and negative events in general (Fivush & Wang, 2005)—authors were unable to find gender-related differences in maternal elaboration. Fivush and Zaman (2014) concluded that activation of gender specific attitudes and behaviors, activation of gender schema is a flexible process. Contexts, such as the age of the child, and the content of the emotional event, activate gender-related cognitions, but other contexts put them into the shade.

To sum up the previous findings we can conclude that narrating emotions is connected to culturally stereotyped gender norms (Grysman & Hudson, 2013) and parents socialize their daughters to focus more on emotions in general and sadness in particular (Fivush & Grysman, 2019). As a result, girls and women report experiencing submissive negative emotions more frequently and intensely than do boys and men (Chaplin & Aldao, 2013). Gender stereotyping studies (Brody, 1999; Plant et al., 2000; Roberts & Goldenberg, 2007) also found that pride was more strongly associated with men than with women. However, Alessandri and Lewis (1993) were unable to observe this gender difference in children as young as 3 years of age. We are ignorant of any investigation of gender differences in mother-child conversations about shame and pride in preschool children. This paucity of research is highly interesting because it is the end of the preschool period when children begin to fully understand self-conscious emotions including shame and pride (Olthof et al., 2000). This is also a salient period of gender and emotion socialization (Brody, 1999; Root & Denham, 2010). In the investigated age range the effect of parental gender role socialization and childrens’ emerging gender stereotyping may amplify small or negligible gender differences in emotion expression of shame and pride (Else-Quest et al., 2012). Based on the above rationale, we designed our study to fill this gap in investigating mother-child conversations about shame and pride in preschoolers.

## Aim of the Study

The major purpose of our study was to examine how mothers socialize their sons and daughters to discuss and understand shame and pride. Previous studies found gender differences in maternal conversational style and emotional content in discussing events related to basic positive and negative emotions (e.g., Fivush et al., 2000). We were interested in whether we can find these differences in conversational style and in the emotional content when mother-child dyads discussed events involving shame and pride that are not basic, but self-conscious positive and negative emotions.

Based on the gender-stereotypes and gender related socialization of the emotions of shame and guilt (see Ferguson & Eyre, 2000), we assumed that mothers would discuss shame related events in more detail (i.e., we expected longer conversations) with daughters than with sons, and use a more elaborative conversational style with daughters than with sons. It means that mothers were expected to give and ask for more new details—elaborative statements—and praise their daughters’ participations with more frequent use of confirmation and repetition of child’s utterances. In addition, we assumed that mother-daughter conversations would be richer in emotional content. Mother-child dyads were expected to use more reference to emotion, emotional behavior and emotional evaluation in shame-conversations, than mothers and sons.

Given the scarce evidence regarding gender differences in experiencing pride, we made no strong predictions, but we assumed that maternal conversational style and emotional content would not show any significant differences between mother-son and mother-daughter pride-conversations. The rationale for formulating this hypothesis comes from the following. If gender differences in shame stories are absent in pride stories, we would consider this to be an evidence for emotion-specific gender differences as opposed to general gender differences in emotion socialization.

Further, Sales and colleagues (2003) found that negative events were discussed in a more elaborative way and with more detailed emotional content. Therefore, as a second goal, we wanted to investigate whether shame-conversations (as recollection of a negative event where the self is negatively appraised) would be more elaborated and contained more references to emotions than pride-conversations (as recollection of a positive event where the self is positively appraised).

## Method

### Participants

Participants were 54 mother-child dyads (28 girls, 26 boys) from low-risk, middle class families living in three mid-sized Hungarian towns. Children were healthy and typically developing. They ranged in age between 50 and 88 months with an average age of 70.36 months (*SD* = 8.13 months). Mothers’ age ranged from 28 to 45 years with an average age of 37.51 years (*SD* = 3.70). Fifty-seven percent of the mothers had high-school education, 43 percent of the mothers had college or university degrees. Mothers and their children mostly lived in two-parent families (9.5% lived in single parent families). Using IBM SPSS (Version 22) for Windows, statistical analyses were done in order to check whether girls and boys differed on any demographic variable. Results of one-way analyses of variance (ANOVAs) and a χ^2^-test revealed that there was no significant difference between girls and boys with regard to their age, *F*(1, 52) = 0.524, *p* = .472, to their mothers’ age, *F*(1, 52) = 1.974, *p* = .166, and to their mothers’ level of education, χ^2^(3) = 1.190, *p* = .755.

Informed consent was obtained from all participating mothers in the study, and no monetary compensation was offered in exchange for participation. However, the children got a small gift after completing the conversations. After completing the conversation both participants were thanked for their time and debriefed. This study was approved by the United Ethical Review Committee for Research in Psychology according to the guidelines of the Hungarian Psychological Society (reference number: 45/2015).

### Procedure

Participants were recruited in seven preschools through flyers and personal contact through preschool teachers to take part in a study about how parents and children discuss the emotional experiences of the child. We used the structured narrative co-construction protocol (Fivush & Grysman, 2019) to get conversations of emotional events. Female research assistants visited the mother-child pairs in their homes. During the home visit, the mother and the child were asked to jointly recall two types of events: one in which the child was ashamed, and one in which the child felt proud. The order of telling the emotional events and the selection of what kind of shame- and pride-events to tell, were decided by the member of the pairs. There were no time restraints and the parents were asked to discuss the events as naturally as if they would if the topic came up in everyday conversations. After informing the mother-child pair about the conversations and how to record the conversations, the assistant left them alone in a room, and asked them to call her when they finished. All conversations were audio recorded and transcribed verbatim for analysis.

### Coding

Coding of the two types of conversations (shame and pride) was conducted in three phases, in order to capture different aspects of the dialogues. The conversations were firstly cleared and only event talk and associative talk—“spinoff” comments related to the event—were involved in later analyses. In the second phase, maternal conversational style was coded using a system adopted from Reese and Fivush (1993). In the third phase, the occurrence of emotional content was counted. The second and third phases are detailed below.

#### Coding Units and Maternal Conversational Style

Event talk and associative talk were segmented into coding units, each consists of a subject-verb construction with a unique or implied verb. The total number of coding units, and the number of maternal and child coding units were counted. In the next step, each coding unit was given an Elaboration or an Evaluation code. Twenty five percent of the conversations were coded by the first author and independent research assistants unaware of research aims. All parent and child coding units were combined across the shame and pride conversations to compute a reliability score for each Elaboration and Evaluation code. Interrater reliability was calculated by intraclass-correlation coefficient (ICC). All discrepancies were discussed between the coders before proceeding. The remaining conversations were coded by the first author. Definitions for coding units and maternal conversational style and coding reliability can be seen in Table 1.

**Table 1:** Definitions and Coding Reliability for Coding Units and Maternal Conversational Style

Coding unit and maternal conversational style code	Definition	Interrater reliability		
		ICC	95% CI
Total number of CU’s	Subject-verb construction with a unique or implied verb (independent clause as proposational unit)	.92	[.90, .94]
Maternal CU’s	Independent clauses told by the mother	.90	[.86, .94]
Child CU’s	Independent clauses told by the child	.93	[.90, .96]
Maternal elaborations			
Open-ended memory questions	Mother asks the child to provide a piece of new memory information about an event	.95	[.93, .97]
Close-ended memory questions	Mother asks questions requiring to confirm or deny a piece of information, or gives a statement including a question tag in order to get confirmation	.92	[.89, .95]
Statement	Mother gives a statement that provides the child with new information	.94	[.90, .98]
Maternal evaluations			
Confirmation	Mother confirms the child previous utterance	.71	[.65, .77]
Repetition	Mother repeats the gist or exact content of a previous utterance of the child	.85	[.81, .89]

*Note*. ICC = intraclass-correlation coefficient; CU = coding units.

#### Emotional Contents

In order to examine how participants explicitly or implicitly referred to emotions, each word referring to specific emotion, emotional behavior and emotional evaluation of the actors or events was counted. Definitions for emotional content and coding reliability can be seen in Table 2.

**Table 2:** Definitions and Coding Reliability for Emotional Contents

Emotional content category/Emotional content subcategory	Interrater reliability		Example
	ICC	95% CI	
Specific emotion			
Positive	.74	[48.65, 92.95]	Happy, was a good feeling
Negative	.78	[48.95, 94.28]	Sad, felt sorry
Emotional behavior			
Positive	.88	[67.48, 96.55]	Laughed, kissed
Negative	.84	[59.67, 95.78]	Cried, hurted
Emotional evaluation			
Positive	.82	[53.70, 95.27]	Of the child: “You were brave”
			Of others’: “They were nice”
Negative	.96	[88.35, 98.89]	Of the child: “You were nasty”
			Of others’: “They were mean”

*Note*. ICC = intraclass-correlation coefficient.

## Statistical Analysis

Two-way mixed effects, single measurement Intraclass Correlation Coefficients (ICCs) were calculated as indices of interrater reliability. Following the guidelines of Portney and Watkins (2000), we interpreted ICC values less than .5 as indicative of poor reliability, values between .5 and .75 as indicative of moderate reliability, values between .75 and .9 as indicative of good reliability, and values greater than .9 as indicative of excellent reliability. Mean (*M*) and standard deviation (*SD*) were calculated for each variable for descriptive purposes. To test the effects of gender, story type, and story type by gender interactions on the frequency of coded content, we used mixed-method ANOVAs. Because each mother-child dyad had conversations about both shame and pride events, we used story type as a within-subject effect. Gender was used as a between-subject effect. In order to further elaborate on results—especially in the case of significant story type by gender interaction, further repeated measure (for comparing shame and pride stories) or one-way (for comparing boys’ and girls’ conversations) ANOVAs were run. Although the level of significance was set at .05, we also report results as marginally significant at the level of .1. Partial η^2^ was used to indicate effect size. According to Cohen (1988), values of .01, .06, and .14 indicate small, medium, and large effects, respectively. Statistical analyses were done with IBM SPSS (Version 22) for Windows.

## Results

First, we calculated ICCs to test interrater reliability of coding. Results are presented in Table 1 and Table 2. According to these results, all codes showed good to excellent reliability, except for Confirmation. The reliability of Confirmation slightly fall below the level of good reliability. This might have been due to coders’ ambiguous interpretation of contentless confirmatory utterances such as “uh-uh” or “uhm.”

Next, we analyzed potential effects of conversation type (shame vs. pride conversations), gender, and conversation type by gender interaction on the emotional content of the stories. To do so, we used repeated measures ANOVAs with conversation type as a within-subject factor and gender as a between-subject factor. Descriptive data for emotional contents’ frequency by conversation type and gender and the results of repeated measures ANOVAs are shown in Table 3.

**Table 3:** Effects of Story Type, Gender and Story Type by Gender Interaction on the Frequency of Emotional Contents—Descriptive Data and Results of Repeated Measures ANOVAs

Emotional content	Shame story		Pride story		Total		*F*(1, 52) (Partial η^2^)		
	*M*	*SD*	*M*	*SD*	*M*_estimated_	*SE*	Story type	Gender	Story type by gender
Specific emotions									
Total							11.253^**^ (.178)	0.780 (.015)	0.500 (.010)
Boys	1.58	1.98	0.69	0.97	1.14	0.24			
Girls	2.11	2.49	0.75	0.93	1.43	0.23
Total	1.85	2.25	0.72	0.94		
Positive							4.598^*^ (.081)	0.259 (.005)	0.207 (.004)
Boys	0.19	0.40	0.58	0.76	0.39	0.09			
Girls	0.32	0.72	0.57	0.84	0.45	0.08
Total	0.26	0.59	0.57	0.79		
Negative							27.385^***^ (.345)	0.631 (.012)	0.378 (.007)
Boys	1.39	1.75	0.12	0.43	0.75	0.21			
Girls	1.79	2.27	0.18	0.48	0.98	0.20
Total	1.59	2.02	0.15	0.45		
Emotional behaviour									
Total							25.792^***^ (.332)	3.755 (.067)	16.082^***^ (.236)
Boys	4.04	3.77	1.00	2.08	2.52	0.41			
Girls	1.61	1.71	1.25	1.48	1.43	0.39
Total	2.78	3.11	1.13	1.78		
Positive							6.337^*^ (.109)	0.320 (.006)	1.211 (.023)
Boys	0.65	1.16	0.96	1.91	0.81	0.21			
Girls	0.25	0.59	1.04	1.37	0.64	0.20
Total	0.44	0.93	1.00	1.64		
Negative							55.331^***^ (.516)	7.128^*^ (.121)	13.329^**^ (.204)
Boys	3.390	2.94	0.04	0.20	1.71	0.25			
Girls	1.36	1.62	0.21	0.57	0.79	0.24
Total	2.33	2.54	0.13	0.44		
Emotional evaluation									
Total							4.124^*^ (.073)	0.121 (.002)	0.323 (.006)
Boys	2.23	1.99	3.50	3.09	2.87	0.35			
Girls	2.68	2.47	3.39	2.51	3.04	0.34
Total	2.46	2.24	3.44	2.78		
Positive							56.131^***^ (.519)	< 0.001 (< .001)	0.808 (.015)
Boys	0.39	0.80	3.39	2.80	1.89	0.28			
Girls	0.71	1.18	3.07	2.34	1.89	0.27
Total	0.56	1.02	3.22	2.55		
Negative							42.094^***^ (.447)	0.408 (.008)	0.029 (.001)
Boys	1.85	1.64	0.12	0.59	0.98	0.18			
Girls	1.96	1.88	0.32	0.72	1.14	0.18
Total	1.91	1.75	0.22	0.66		

*p < .05. **p < .01. ***p < .001.

With regard to specific emotions, pride conversations contained significantly more positive specific emotions than shame conversations. Shame conversations contained significantly more negative specific emotions than pride stories. The frequency of overall specific emotions was higher in shame stories than in pride stories. From the three above-mentioned results, we might infer that shame stories contained significantly more negative specific emotions compared to positive specific emotions in pride stories. The result of a repeated measures ANOVA supported the above inference, *F*(1, 53) = 11.736, *p* < .005, partial η^2^ = .181; for means and *SD*s see Table 3.

With regard to emotional behavior, a significant story type by gender interaction effect was found on the frequency of emotional behaviors in total and for negative emotional behaviors as well. To be able to better interpret these interactions we used repeated measures ANOVAs separately for the subsamples of boys and girls. We found that boys’ stories of shame contained significantly more overall emotional behaviors than their stories of pride, *F*(1, 25) = 25.759, *p* < .001, partial η^2^ = .507; for means and *SD*s see Table 3. For girls, stories of shame didn’t significantly differ from stories of pride with respect to total frequency of emotional behaviors, *F*(1, 27) = 1.199, *p* = .283, partial η^2^ = .043; for means and *SD*s see Table 3. Using the same statistical analyses for negative emotional behaviors revealed that the above-mentioned interaction effect on overall emotional behaviors was predominantly due to negative emotional behaviors. Stories of shame contained significantly more negative emotional behaviors than their stories of pride for boys as well as for girls, *F*(1, 25) = 37.154; *p* < .001; partial η^2^ = .598 for boys; *F*(1, 27) = 16.615, *p* < .001, partial η^2^ = .381 for girls; for means and *SD*s see Table 3. Effect size was smaller for girls. Analyzing the effect of gender on negative emotional behaviors with ANOVAs separately for shame and pride stories revealed what follows. Whereas the amount of negative emotional behaviors was the same in pride stories for boys and for girls, *F*(1, 52) = 2.240, *p* = .141; for means and *SD*s see Table 3, shame stories of boys contained significantly more negative emotional behaviors than the shame stories of girls, *F*(1, 52) = 10.055, *p* < .005; for means and *SD*s see Table 3. With respect to positive emotional behaviors, pride stories contained significantly more positive emotional behaviors than shame stories.

With regard to emotional evaluations, pride stories contained significantly more positive emotional evaluations than shame stories. Shame stories contained significantly more negative emotional evaluations than pride stories. The frequency of overall emotional evaluations was higher in pride stories than in shame stories. From the three above-mentioned results, we might infer that pride stories contained significantly more positive emotional evaluations compared to negative emotional evaluations in shame stories. The result of a repeated measures ANOVA supported the above inference, *F*(1, 53) = 9.237, *p* < .005, partial η^2^ = .148; for means and *SD*s see Table 3.

Next, we analyzed the potential effects of story type (shame vs. pride stories), gender, and story type by gender interaction on the frequency of coding units and indices of maternal conversational style with the same statistical procedure as described above. Descriptive data for the frequency of coding units and indices of maternal conversational style by story type and gender along with the results of repeated measures ANOVAs are shown in Table 4.

**Table 4:** Effects of Story Type, Gender and Story Type by Gender Interaction on the Frequency of Coding Units and Maternal Conversational Style Codes—Descriptive Data and Results of Repeated Measures ANOVAs

Coding unit and maternal conversational style code	Shame story		Pride story		Total		*F*(1, 52) (Partial η^2^)		
	*M*	*SD*	*M*	*SD*	*M*_estimated_	*SE*	Story type	Gender	Story type by gender
Coding units									
Total							3.235^#^ (.059)	4.1157^*^ (.074)	2.331 (.043)
Boys	48.23	21.06	47.35	24.93	47.79	3.59			
Girls	43.04	25.64	32.21	14.03	37.63	3.46
Total	45.54	23.47	39.50	21.25		
Mother							3.655^#^ (.066)	2.601 (.048)	1.607 (.030)
Boys	30.69	14.88	29.23	17.16	29.96	2.71			
Girls	27.50	20.84	20.29	9.50	23.89	2.61
Total	29.04	18.12	24.59	14.33		
Child							1.828 (.034)	6.287^*^ (.108)	3.128^#^ (.057)
Boys	17.58	8.05	18.12	10.03	17.85	1.35			
Girls	15.18	8.34	11.14	6.74	13.16	1.30
Total	16.33	8.21	14.50	9.11		
Maternal elaborations									
Open-ended memory questions							0.073 (.001)	4.172^*^ (.074)	0.296 (.006)
Boys	5.23	3.45	5.65	4.61	5.44	0.62			
Girls	3.75	3.33	3.61	3.32	3.68	0.60
Total	4.46	3.44	4.59	4.09		
Close-ended memory questions							2.496 (.046)	3.004^#^ (.055)	0.225 (.004)
Boys	7.85	6.23	6.92	5.04	7.39	0.80			
Girls	6.32	5.10	4.61	3.80	5.46	0.77
Total	7.06	5.67	5.72	4.55		
Statements							1.081 (.020)	0.085 (.002)	0.350 (.007)
Boys	6.77	7.37	6.31	5.21	6.54	1.02			
Girls	6.96	8.11	5.29	4.25	6.13	0.98
Total	6.87	7.69	5.78	4.72		
Maternal evaluations									
Confirmations							1.024 (.019)	0.025 (< .001)	1.985 (.037)
Boys	0.81	1.27	1.46	2.37	1.14	0.28			
Girls	1.25	1.71	1.14	1.41	1.20	0.27
Total	1.04	1.52	1.30	1.92		
Repetitions							1.757 (.033)	6.186^*^ (.106)	0.178 (.003)
Boys	2.46	2.30	2.23	1.90	2.35	0.30			
Girls	1.54	1.35	1.11	1.40	1.32	0.29
Total	1.98	1.91	1.65	1.74		

^#^*p* < .1. *p < .05.

According to the results with respect of the length of conversations (as indicated by the frequency of coding units), shame stories were marginally significantly longer and contained marginally significantly more maternal contributions than pride stories. Stories of boys were significantly longer than stories of girls. A marginally significant story type by gender interaction effect on the length of children’s contributions was revealed. Comparing length of child contributions separately in the subsamples of boys and girls with repeated measures ANOVAs showed what follows. For boys, the length of their own contributions didn’t significantly differ in shame and pride stories, *F*(1, 25) = 0.062, *p* = .806, partial η^2^ = .002; for means and *SD*s see Table 4. Girls made significantly shorter contributions to pride stories than to shame stories, *F*(1, 27) = 7.540, *p* < .05, partial η^2^ = .218; for means and *SD*s see Table 4.

With respect to maternal elaborations, mothers used significantly more open-ended memory questions and marginally significantly more close-ended memory questions with boys than with girls. With regard to maternal evaluations, mothers used significantly more repetitions with boys than with girls. Neither story type nor the story type by gender interaction had a significant effect on maternal elaborations and evaluations.

## Discussion

In this study, we explored how mothers discuss shame experiences and pride experiences with their 4–6 years old daughters and sons. The results indicated gender differences in some aspects of the conversations, although in the opposite direction than it was expected. Mothers generally tended to discuss both self-conscious emotions in a more elaborated way with their sons than with their daughters. Effects of the conversation type (shame vs. pride) were also found, but the effects of conversation type and gender were in mixed directions depending on the outcome variable.

First, we found that shame conversations contained more negative specific emotions, and pride conversations contained more positive specific emotions. This result was not surprising because words referring to specific emotions might have reflected the positive and negative emotional valence of pride and shame stories, respectively. Former studies also found higher proportion of positive emotion than negative emotion in the positive event conversations, and higher proportion of negative emotion than positive emotion in negative event conversations (e.g., Sales et al., 2003). These results also confirmed that the prompting of the stories was successful: the dyads discussed more positive feelings related to pride, and more negative feelings related to shame. More interestingly mothers and children told more specific emotions in total (i.e., independent of valence) in shame stories than in pride stories. Compared to positive emotion words in pride stories the number of specific negative emotion words was higher in shame stories. This result corresponds with previous results of mother-child conversations about negative and positive events, which found that the discourse between mothers and children in the negative event conversations was richer in emotions than in the positive event conversations (e.g., Laible, 2011; Sales et al., 2003). Although in Sales et al.’s (2003) or Laible’s (2011) studies the emotional richness reflected more references to causes of emotions in the negative stories, and the number of emotional terms was not higher in negative stories, in our study participants referred more frequently to specific emotions—especially to specific negative emotions –in shame stories. According to Edelstein and Shaver’s (2007) review about the lexical representation of self-conscious emotions, in Western individualistic cultures shame is categorized as either a subcategory of sadness or fear as superordinate emotions. The representation of shame depends on the culture’s emphasis on shame as an experience and whether it is linked more with the individual’s negative affections—regret, remorse—after doing something inappropriate or with the anxiety or ambivalence about committing a particular transgression. It could be argued that in discussing events related to negative self-conscious emotions, mothers and children may refer to negative emotions associated to shame-experience to understand the negative aspects of shame. Basic emotions of sadness and fear are conceived of as developmentally earlier than self-conscious emotions (e.g., Bretherton & Beeghly, 1982), and children between 3–5 years are already able to categorize pride with positive emotions and shame, guilt, and embarrassment with negative emotions (e.g., Lagattuta & Thompson, 2007). Mothers may scaffold the child’s emerging emotional understanding by anchoring the feeling of shame and guilt to other salient negative emotions—such as sadness and fear.

Here it should be noted that prompting shame-conversations was at one side successful, because mother-child dyads talked about events that were related to negative self-conscious emotions, but on the other hand, most of the conversations were rather about guilt than shame. The topics of shame/guilt events were mostly breaches of decorum (e.g., saying naughty/dirty words); hurting others; damaging objects; and in a smaller extent threat of unwanted identity in face of peers (e.g., unintentional soiling; failure in peer activity). It is not surprising however, if we consider the age of the children. In the preschool years, shame and guilt are intermingled in experience, and children have difficulties in differentiating them (Olthof at el., 2004). Shame-stories in our study therefore were more often associated to moral transgressions than to the child’s appraisal of the self as fundamentally defective (Lewis, 1971). Therefore, sadness caused for example by the child’s aggressive behavior and fear of expected punishment and/or fear of rejection by the parent occurred as well. On the other hand, it should also be taken into account that parents report using conversations about emotions to facilitate the acquisition of social norms (Kulkofsky, Wang, & Koh, 2009). Perhaps guilt-events were more salient for mothers and children, and discussing moral transgressions might have been emotionally less painful. It could have been also easier for the mother to perceive the child’s guilt that could have been linked to more transparent breaches of moral norms, than to perceive the child’s shame that could have been associated with the mothers’ own disapproval or rejection of the child, or with the child’s experience of shame in face of peers—which is usually more difficult for mothers to perceive.

With regard to emotional behaviors, we found that shame stories contained more negative emotional behaviors than the stories of pride, irrespective of the child’s gender. Further, shame stories of boys contained significantly more negative emotional behaviors than the shame stories of girls. Tangney and Dearing (2002) in their review concluded that when describing personal shame and guilt experiences preschool aged children were more likely to mention visible and concrete behaviors, such as hitting a sibling, damaging an object, or disobeying parents, than less visible failures and transgressions. This is in line with our results, because in our study negative emotional behaviors referred to visible negative behavioral antecedents of shame/guilt (e.g., hurting someone) and the consequences of breaking the rules (e.g., parental physical punishment). The results also showed that this emphasis on the behavioral aspects of shame/guilt is more intense in boys than in girls. With regard to triggering events, mothers and sons identified aggressive moral transgressions as leading to shame/guilt more frequently. With regard to consequences, the results could have also reflected the more intense externalization tendencies of boys. As a reaction to shame/guilt, they showed more anger than girls—as previously described by Muris and Meesters (2014)—and this anger was controlled by the parent in the case of sons at the behavioral level as well. Parents with traditional attitudes toward gender roles usually use physical punishment more frequently with boys (Endendijk, Groeneveld, Bakermans-Kranenburg, & Mesman, 2016) and this could have been expressed by the greater number of negative emotional behavior words in the shame-stories of boys. Alternatively, this result can be explained by previous findings that pointed to the fact that anger as a basic emotion was discussed more deeply and in a more detailed way with boys than with girls (e.g., Bird & Reese, 2006). As previously mentioned, anger was intensely related to shame/guilt in most of the conversations in our study.

With regard to emotional evaluations, also these results reflected the emotional valence of the stories: pride stories contained significantly more positive emotional evaluations than shame stories. Interestingly however, the frequency of overall emotional evaluations was higher in pride stories than in shame stories, and pride stories contained significantly more positive emotional evaluations compared to negative emotional evaluations in shame stories. This might suggest that whereas the experience of shame/guilt could be best elaborated by the help of referring to the internal state of the experiencer and to behaviors that elicit or follow shame/guilt, negative evaluations are avoided by parents. This could be the result of parents’ willingness to protect the child’s face and the parent-child relationship in the conversations by the use of less negative appraisals in shame/guilt stories. Expanding the meaning of shame/guilt experiences by the means of devaluing negative emotional evaluations would humiliate the child in the reminiscing situation. This speculation is in line with the general assumption about the aversive nature of shame/guilt experiences in most European cultures (Edelstein & Shaver, 2007). The other side of this emotionally supportive parental attitude could have been represented in the result that parents used more frequent positive evaluations—praise—when expanding the meaning of pride. This corresponds with the major theories of socialization of pride that point to the role of parental praise and approval (Lagattuta & Thompson, 2007). Consequently, it can be speculated that in shame/guilt stories the use of specific emotion words and references to emotional behaviors serve the processes of emotional understanding. Negative emotional evaluations may be more related to the affective aspects of the parent-child relationship, therefore parents use them more cautiously in elaborating on shame/guilt events.

Considering the length of conversations, we found that shame stories were marginally significantly longer and contained marginally significantly more maternal contributions than pride stories. Talking more about an emotion could implicate that the members of the dyad—and especially the mother—find it important to learn/teach more about nature of shame/guilt and how to cope with these feelings (Fivush et al., 2000). This process of teaching/learning about emotions could be especially significant for boys, since we found that stories of sons were significantly longer than stories of daughters, irrespective of the emotion under discussion, and it was due to greater participation of sons (and not mothers). We might argue that mothers grabbed the opportunity—as expressed by more maternal elaboration and evaluation as discussed later—to talk about emotion-laden events with their sons in this laboratory situation. If future research proves this suggestion to be valid, it could mean that boys are at least as open to discuss emotions as girls. Previously observed gender differences (e.g., Fivush et al., 2003) might rather be interpreted in terms of motivation or adherence to social norms than in terms of socioemotional competencies. We might speculate that in adhering to social expectations, mothers—and maybe their sons—actively avoid situations where emotions could be discussed.

A marginally significant story type by gender interaction showed that boys made the same amount of contribution to shame stories as to pride stories, whereas girls made significantly shorter contributions to pride stories than to shame stories. Investigating girls and boys display of shame related behavior, Alessandri and Lewis (1993) found that 3–6 year-old girls displayed more shame-relevant behaviors after failure. If this means that girls are socialized more to experience shame/guilt and are more skilled in expressing them in daily interactions, there is no need to an intense support of teach about the emotions in the emotional conversations, as it is in the case of boys. A similar gender specific tendency can be observed in pride stories, in which boys’ contribution was significantly higher than girls’. Brody (1999) in her review summarized findings in which men report more pride and this may reflect their higher status and higher self-esteem. We could argue that former socialization experiences of boys lead them to show a more intense participation in pride-conversations, because they are taught to be more skilled to verbalize pride experiences, than girls.

With respect to maternal elaborations and evaluations, we found that mothers used significantly more open-ended memory questions and marginally significantly more close-ended memory questions with boys than with girls, and mothers used significantly more repetitions with boys than with girls. This means that irrespective of the emotion discussed, mothers provide more structured questions and use more motivation with boys than with girls. This is in line with Sales et al.’s (2003) findings and we share their assumption that boys’ decreased willingness and/or capacity to participate in reminiscing might make parents use this more directive and motivating narrative style to keep their sons engaged in conversation.

Besides the tempting results, several limitations of the study must be acknowledged.

Language and communication are highly influenced by sociocultural and sociodemographic background (Otto, 2006). Results of statistical analyses further confirmed that girls and boys did not significantly differ with regard to their own age and to their mothers’ age or level of education. Thus, our homogenous sample made it possible to draw specific conclusions about the role of the gender per se. However, the homogeneity of our sample prevents us to generalize our findings to mother-child dyads coming from different sociodemographic background. Further studies should clarify whether our findings apply to dyads from, for example, economically disadvantaged environments or from psychologically vulnerable groups, for example, children at risk for mental disorders.

Emotions and their functions are interpreted in different ways in different cultures, thus, the cross-cultural generalizability of our findings is highly questionable. Although cultural differences in the meaning of shame and guilt in other European languages have been already published (e.g., Olthof et al., 2004 for Dutch), there has not been any research about the cognitive representation of shame and guilt in Hungarian, yet. In the everyday practice when parents in Hungary use the formula “Ezért most szégyellheted magad!” (“You should be ashamed because of this!”) the preceding event is a violation of an external standard, and the parent intend to induce negative self-conscious emotion relevant to the situation—whether it should be shame or guilt. The conceptual overlap of shame, embarrassment and guilt in Hungary could have affected how children and mothers interpreted the instruction and chose the event to tell.

The function that parents attribute to parent-child conversations about past events is culturally varied as well. Zevenbergen, Haman, and Olszańska (2012) found significant differences between East-European (Polish) and US mothers’ reports on the reasons of reminiscing. Polish mothers emphasized the differentiation of morally Good from morally Bad in the Polish sample. Presumed the cultural and historical similarities between Hungary and Poland, we suppose that our results could be influenced by these underlying beliefs about the functions of emotional reminiscing.

Although previous studies with similar paradigm analyzed parental and child conversational style separately, we decided to analyze maternal conversational style only. We did this because children’s participation in conversations at this age is highly organized and influenced by adults’ scaffolding (Haden, Haine, & Fivush, 1997). Thus, we assumed that children’s conversational style would rather be the reflection of maternal conversational style than the independent characteristic of the children themselves. Further studies should check whether this assumption could be empirically supported.

Another limitation could be the fact that we coded emotional contents in general, irrespective of the source of contribution. Because maternal and child contributions were not coded separately, it is difficult to determine which partner’s participation contributed more to the results. At the same time, it depends on the outcome of the conversation—that is a bidirectional and reciprocal activity—how the child organizes her knowledge about experiencing and expressing an emotion (Laible, 2004a). Therefore, it might make more sense to code and interpret emotional content irrespective of contributor, as we did it in our study.

Last—but not least—only mother-child dyads and no father-child dyads participated in our study. Although in the Hungarian culture—just like in other societies with more conservative gender ideologies—mothers are more intensely involved in socialization processes (Murinkó, 2014), we are reluctant to conclude that a replication of this study with fathers would lead to the same results. What makes us reluctant is the fact that the gendered nature of emotion socialization might not only affect children but parents as well. Future research should take not only child gender, but parent gender into account as well.

Investigation of verbal socialization of shame and pride helps us to understand the sociocultural aspects of the development of self-conscious emotions and the individual differences in proneness to shame and proneness to pride. Since shame and pride are self-evaluative emotions and both are related to the vicissitudes of self-esteem their investigation has an implication for mental health.
